# Beneficial effects of antioxidative lactic acid bacteria

**DOI:** 10.3934/microbiol.2017.1.1

**Published:** 2017-01-06

**Authors:** Hisako Nakagawa, Tadaaki Miyazaki

**Affiliations:** Department of Probiotics Immunology, Institute for Genetic Medicine, Hokkaido University, N15, W7, Kita-ku, Sapporo, Japan

**Keywords:** lactic acid bacteria, antioxidant, oxidative-stress related disease, aging and prolongevity

## Abstract

Oxidative stress is caused by exposure to reactive oxygen intermediates. The oxidative damage of cell components such as proteins, lipids, and nucleic acids one of the important factors associated with diabetes mellitus, cancers and cardiovascular diseases. This occurs as a result of imbalance between the generations of oxygen derived radicals and the organism's antioxidant potential. The amount of oxidative damage increases as an organism ages and is postulated to be a major causal factor of senescence. To date, many studies have focused on food sources, nutrients, and components that exert antioxidant activity in worms, flies, mice, and humans. Probiotics, live microorganisms that when administered in adequate amounts provide many beneficial effects on the human health, have been attracting growing interest for their health-promoting effects, and have often been administered in fermented milk products. In particular, lactic acid bacteria (LAB) are known to conferre physiologic benefits. Many studies have indicated the antioxidative activity of LAB. Here we review that the effects of lactic acid bacteria to respond to oxidative stress, is connected to oxidative-stress related disease and aging.

## 1. Introduction

Oxidants are generated as a result of normal intracellular metabolism in mitochondria and peroxisomes, as well as from a variety of cytosolic enzyme systems, in addition, a number of external agents trigger reactive oxygen species ROS production. ROS can be produced endo- or exogenously. The level of ROS is regulated by antioxidants defense mechanisms. In this way, the cell encases an antioxidant/pro-oxidant balance [1]. Organisms have developed an overall antioxidative defense system to mitigate the damaging effects of ROS. To protect against oxidative stress, eukaryotes possess sophisticated defense systems that cope with elevated ROS levels and promote homeostasis [2]. Proteins that protect against high ROS levels include catalases, superoxide dismutases (SODs), and glutathione peroxidases (GSH-Pxs), and these signaling pathways are known to be strongly evolutionarily conserved. Lowering ROS levels below the homeostatic set point may interrupt the physiological role of oxidants in cellular proliferation and host defense. Similarly, increased ROS may also be detrimental and lead to cell death or to an acceleration in ageing and age-related diseases. Many environmental stimuli including produced inflammatory cytokines, ultraviolet radiation, smoking, chemotherapeutic agents and even growth factors generate high levels of ROS that can perturb the normal redox balance and shift cells into a state of oxidative stress. Traditionally, the impairment caused by increased ROS is thought to result from random damage to proteins, lipids and DNA. In addition to these effects, a rise in ROS levels may also induce a stress signal that activates specific redox-sensitive signaling pathways. Once activated, these diverse signaling pathways may have either damaging or potentially protective functions [2]. These defence systems are not effective enough to totally prevent the damage, and therefore, food supplements containing antioxidants may be used to help the human body to reduce oxidative damage [3],[4],[5] . In several decades, many studies have focused on food sources, nutrients, and components that exert an inhibitory effects on the antioxidative activity in humans and other animals. It has been shown that some lactobacilli possess antioxidant activity, and are able to decrease the risk of accumulation of ROS during ingestion of food [6].

Lactic acid bacteria (LAB) is well known as “Probiotics”. Probiotics have been defined as “live microorganisms, which when administered in adequate amounts, confer a beneficial health effect on the host [7].” LAB are Gram-positive bacteria, widely distributed in nature, and industrially important as they are used in a variety of industrial food fermentations. The potential benefits of LAB for human and animal health include stimulation of the immune system, balancing intestinal flora, and reducing serum cholesterol. Lately, some LAB strains have been found with other important biological functions, such as anti-ageing and antioxidant activities. We review that the effects of LAB to respond to oxidative stress, is connected to oxidative-stress related disease and aging.

## 2. The Effects of LAB on the Prevention of Diseases Related to Oxidative Stress

*Lactobacillus rhamnosus* GG was found to inhibit lipid peroxidation in vitro due to iron chelation and superoxide anion scavenging ability [8]. In systems mimicking colon fermentation, *Lactobacillus paracasei* Fn032, *Lactobacillus rhamnosus* GG and *Lactobacillus* spp Fn 001 have been shown to prevent hydroxyl radical production [9]. Moreover, it has been shown that orally-administered live recombinant LAB producing bacterial SOD can improve TNBS-induced colitis in rats [10],[11]. And Grompone et al. reported that *L. rhamnosus* CNCM I-3690 has a strong anti-inflammatory profile in co-culture with intestinal epithelial cell-lines, in vitro and this was confirmed in a TNBS-induced colitis model in mice [12]. Guo et al. showed that expolysaccharide of *Lactococcus lactis* subsp. exhibited antioxidant activity, as shown by evaluation of CAT, SOD and GSH-Px activity, as well as MDA levels in blood serum and the livers of mice [13]. Increasing oxidative stress in accumulated fat is an important pathogenic mechanism of obesity-associated metabolic syndrome. The role of oxidative stress in the pathophysiologic interactions among the constituent factors of the metabolic syndrome has been remarked. Epidemiological, clinical, and animal studies have shown that obesity is coupled with altered redox state and increased metabolic risk. *Lactobacillus fermentum* ME-3 possessed Mn-superoxide dismutase activity and both its lysates and intact cells were capable of increasing the glutathione redox ratio in blood sera, and improving the composition of the low-density lipids and post-prandial lipids [14]. *Lactobacillus casei* *Zhang* was shown to alleviate oxidative stress by reducing lipid peroxidation and improving lipid metabolism both in blood and liver [15]. *Lactobacillus plantarum* 7FM10 exhibited DPPH and superoxide radical scavenging capacities [16]. Amaretti et al. groups [17] reported that the strains *Bifidobacterium animalis subsp. lactis* DSMZ 23032, *Lactobacillus acidophilus* DSMZ 23033, and *Lactobacillus brevis* DSMZ 23034 exhibited among the highest antioxidants activity within the lactobacilli and bifidobacteria. Park et al. indicated the possibility that probiotic treatment reduce diet-induced obesity and modulate genes associated with metabolism and inflammation in the liver and adipose tissue [18]. Therefore, the effects of antioxidative LAB crosstalk between metabolism and inflammatory signaling pathways.

## 3. Free Radical Theory for the Process of Aging

Aging induced by the accumulation of molecular damage, cellular dysfunction, and reduced functioning of organs for the entire lifetime, often leads to frailty, malfunction and lifestyle-related diseases. Dr. Harman articulated a “free-radical theory” of ageing, speculating that endogenous oxygen radicals were generated in cells and resulted in a pattern of cumulative damage [19]. To protect against oxidative stress, eukaryotes possess sophisticated defense systems that cope with elevated ROS levels and promote homeostasis. Hallmarks of aging include genomic instability, telomere attrition, epigenetic alteration, loss of proteostasis, deregulated nutrient sensing, mitochondrial dysfunction, and cellular senescence [20]. To date, many studies have focused on food sources, nutrients, and components that exert inhibitory effects on the hallmarks of aging in worms, flies, mice, and humans. In 1907, Dr. Metchnikoff first proposed the concept of probiotic bacteria, hypothesizing that lactobacilli were important for promoting human health and longevity [21] and that consumption of lactic-acid-producing bacteria [22], such as the lactobacilli found in yogurt, could be useful for prevention of aging and extension of lifespan. The mechanisms behind the probiotic effects of bacteria, however, are not entirely understood.

Recently, some groups reported the action mechanism by LAB for longevity by using *Caenorhabditis elegans* (*C.elegans*). *C.elegans* is possibly the most suitable model organism for research on the mechanism of the process for aging. The reason is that it has an evolutionarily conserved metabolism and host defense mechanisms, including insulin/insulin-like growth factor (IGF-1) signaling pathway [23], p38 mitogen-activated protein kinase (p38 MAPK) pathway [24], and the transforming growth factor (TGF-) signaling pathway [25]. Moreover, dietary resources, such as bacteria, play an important role in the control of the lifespan of *C. elegans* [26]. Aging in *C.elegans* is a complex process driven by diverse molecular signaling pathways.

Many genes that are differentially regulated in young versus old animals are known or postulated to be regulated by DAF-16 [forkhead box O (FOXO) transcription factor] [23],[27] and SKN-1 [ortholog of mammalian NF-E2-related factor 2 (NRF2)] [28],[29]. DAF-16 and SKN-1 play highly conserved roles in regulating stress resistance and longevity genes. Grompone et al. showed that *Latobacillus rhamnosus* CNCM I-3690 exerted a strong antioxidant effect and extended nematode lifespan through the insulin-like pathway DAF-2/DAF-16 [12]. On the other hand, we recently, found that feeding with *Lactobacillus gasseri* SBT2055 (LG2055) prolonged the lifespan of *C. elegans* compared with that with the control *E. coli*. We also observed that the feeding for *C. elegans* mutants, *daf-2* (e1368) and *daf-16* (mgDf50) with LG2055 extended their lifespan similarly to that for the wild-type worms. In contrast, the feeding with LG2055 did not extend the lifespan of skn-1 mutant worms [30]. Therefore, LG2055 was demonstrated to prolong the worm lifespan through the regulation of SKN-1. SKN-1 plays physiological regulatory roles in multiple processes, including detoxification, metabolism, the immune response, and the oxidative-stress defense.

The maintenance of low ROS levels is critical for function of normal cell function. Thus, we also investigated whether LG2055 stimulated the host defense system and ROS production. Hoeven et al. have shown that ROS released from Ce-Duox1/BLI-3 can activate SKN-1 activity via p38 MAPK signaling [31], with NSY-1 and SEK-1 both able to regulate the p38 MAPK ortholog PMK-1. In response to oxidative stress, PMK-1 phosphorylates SKN-1, which then translocates to the nuclei of intestinal cells and induces the transcription of phase 2 detoxification genes [32]. The p38 MAPK pathway is also known to be crucial for stress response and regulation of immunity. Papp et al. showed that SKN-1 and PMK-1 were central elements in immunosenescence [33]. Immunosenescence, or the age-dependent decline in immune responsivity, is a critical condition that impedes healthy aging [34]. We found that feeding with LG2055 effectively stimulated NSY-1-SEK-1-PMK-1-SKN-1 signaling pathway. Ikeda et al. [35] have also studied the effects of different probiotic strains in *C. elegans*, including bifidobacterium, *Lactobacillus helveticus*, and *Lactobacillus plantarum*. Immune-stimulating molecules, such as peptidoglycan [36], S-layer protein [37], and expolysaccharide [38],[39],[40] exist on the cell surfaces of these bacteria. Therefore, the beneficial efficacy of LAB may be influenced by differences in the structures of immune-stimulating molecules. In addition, the antioxidants LAB continue to be isolated from traditional fermented food and the intestine of marine organism [41],[42]. In the future, some other as yet unknown factors could be shown to be critical for the regulation of immunity.

In conclusion, the significant antioxidative activity is the basis for the increased resistance of LAB to toxic oxidative compounds and helps some isolates of Lactobacillus spp. to serve as defensive components in intestinal microbial ecosystem. Such antioxidative bacteria strains, with desirable properties, should be a promising material for both applied microbiology and scientific food industry, considering the fact that human microbiota have to be tolerant to endogenous and exogenous oxidative stress to prevent or treat many human diseases.
